# A New Essential Oil from the Leaves of *Gynoxys rugulosa* Muschl. (Asteraceae) Growing in Southern Ecuador: Chemical and Enantioselective Analyses

**DOI:** 10.3390/plants12040849

**Published:** 2023-02-14

**Authors:** Yessenia E. Maldonado, Omar Malagón, Nixon Cumbicus, Gianluca Gilardoni

**Affiliations:** 1 Departamento de Química, Universidad Técnica Particular de Loja (UTPL), Calle Marcelino Champagnat s/n, Loja 110107, Ecuador; 2 Departamento de Ciencias Biológicas y Agropecuarias, Universidad Técnica Particular de Loja (UTPL), Calle Marcelino Champagnat s/n, Loja 110107, Ecuador

**Keywords:** gas chromatography, mass spectrometry, enantiomers, 2,3-diacetyl-6-*tert*-butyldimethylsilyl-β-cyclodextrin, 2,3-diethyl-6-*tert*-butyldimethylsilyl-β-cyclodextrin

## Abstract

An essential oil, distilled from the leaves of the Andean species *Gynoxys rugulosa* Muschl., is described in the present study for the first time. The chemical composition was qualitatively and quantitatively determined by GC–MS and GC–FID, respectively. On the one hand, the qualitative composition was obtained by comparing the mass spectrum and the linear retention index of each component with data from literature. On the other hand, the quantitative composition was determined by calculating the relative response factor of each constituent, according to its combustion enthalpy. Both analyses were carried out with two orthogonal columns of nonpolar and polar stationary phases. A total of 112 compounds were detected and quantified with at least one column, corresponding to 87.3–93.0% of the whole oil mass. Among the 112 detected components, 103 were identified. The main constituents were α-pinene (5.3–6.0%), (*E*)-β-caryophyllene (2.4–2.8%), α-humulene (3.0–3.2%), germacrene D (4.9–6.5%), δ-cadinene (2.2–2.3%), caryophyllene oxide (1.6–2.2%), α-cadinol (3.8–4.4%), 1-nonadecanol (1.7–1.9%), 1-eicosanol (0.9–1.2%), *n*-tricosane (3.3–3.4%), 1-heneicosanol (4.5–5.8%), *n*-pentacosane (5.8–7.1%), 1-tricosanol (4.0–4.5%), and *n*-heptacosane (3.0–3.5%). Furthermore, an enantioselective analysis was carried out on the essential oil, by means of two cyclodextrin-based capillary columns. The enantiomers of α-pinene, β-pinene, sabinene, α-phellandrene, β-phellandrene, linalool, α-copaene, terpinen-4-ol, α-terpineol, and germacrene D were detected, and the respective enantiomeric excess was calculated.

## 1. Introduction

During the last 40 years, the phytochemical investigation has shifted from temperate climates to tropical countries, where most of the botanical species are still unstudied [1,2]. In this sense, a great importance is given to the so-called “megadiverse” countries, a group of 17 countries, including Ecuador, characterized by possessing three-fourths of all higher plant species of the world [3]. For this reason, our group has been investigating the phytochemistry of the Ecuadorian flora for more than 20 years, by describing the major metabolites of unprecedented botanical species [4,5,6,7,8,9]. Together with nonvolatile compounds, we are very interested in essential oils (EOs), defined by the European Pharmacopoeia as “odorous products, usually of complex composition, obtained from a botanically defined plant raw material by steam distillation, dry distillation, or a suitable mechanical process without heating” [10,11,12,13,14,15,16,17]. Our interest in the EOs derives from the commercial importance of these mixtures and, overall, from the fact that they can be sources of new or rare sesquiterpenoids, often biologically active, together with enantiomeric compounds. As discussed in a previous paper, we selected the poorly studied genus *Gynoxys* as a promising taxon for a systematic investigation. Despite the leaves not usually being very fragrant, a preliminary unpublished analysis indicated that the EOs from this genus are dominated by the sesquiterpene fraction [18]. For what concerns *Gynoxys rugulosa* Muschl., this species is poorly described also from the botanical point of view. In fact, on the one hand, it is not present in the Catalog of the Vascular Plants of Ecuador. On the other hand, the online database Tropicos only reports three specimens for this plant, from northern Peru and southern Ecuador, where the species grows at an altitude of 2500–3000 m above the sea level [19].

**Figure 1:**
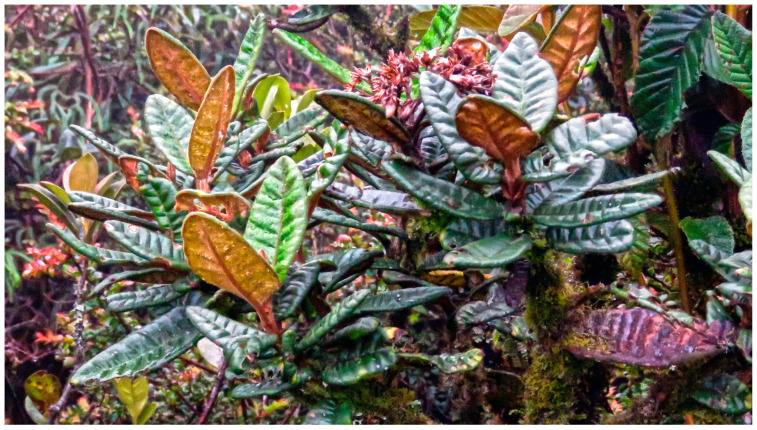
*G. rugulosa* shrub at the collection site (photo by G. Gilardoni).

Botanically (see Figure 1), *G. rugulosa* is a shrub growing up to 2 m tall, with compressed-quadrangular branches and tomentosa, blackish brown in color. The leaves are opposite and petiolate, with an acute apex, rounded, or sometimes subcordate-rounded base, and yellowish tomentose underside, with pinnate venation. The plant presents sub corymbose, compound inflorescence at the apex of the terminal branches. This species only grows in shrubby Paramos, sharing the same ecosystem of typical families such as Melastomataceae, Asteraceae, Orchidaceae, and Ericaceae [20].

Since this plant is little known and quite rare, no traditional use exists to the best of the authors’ knowledge. From the legal point of view, probably due to the lack of botanical information, *G. rugulosa* is not a protected species, and it does not even appear in the reference publication for threatened taxa (The Red Book of the Endemic Plants in Ecuador). Therefore, the present study presents the first description of an EO distilled from *Gynoxys rugulosa* Muschl., together with the enantiomeric composition of some chiral terpenes.

## 2. Results

### 2.1. Chemical Analysis of the EO

The detailed amount of each component and fraction is represented in Table 1. Overall, with respect to the polar and nonpolar column, the monoterpene fraction ranged between 12.9% and 10.3% of the whole EO respectively, the sesquiterpene fraction ranged between 39.1% and 43.3%, and the other non-terpene compounds ranged between 35.3% and 39.4%. A total of 87.3–93.0% of the oil mass was quantified. The distillation yield of this EO, analytically calculated over four repetitions, was 0.02% ± 0.004% by weight of dry plant material.

**Figure 2:**
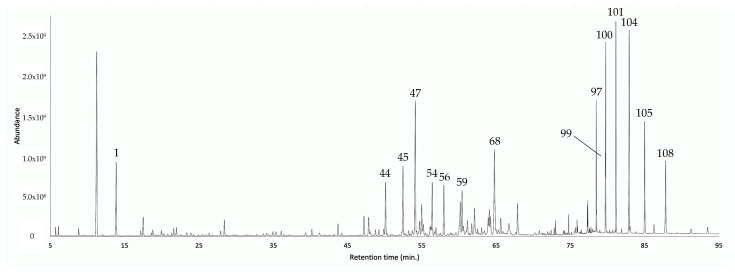
GC–MS chromatogram of the EO from the leaves of *G. rugulosa* in a 5% phenyl-methylpolysiloxane-based column. The main components are represented with the respective peak number, according to Table 1.

**Figure 3:**
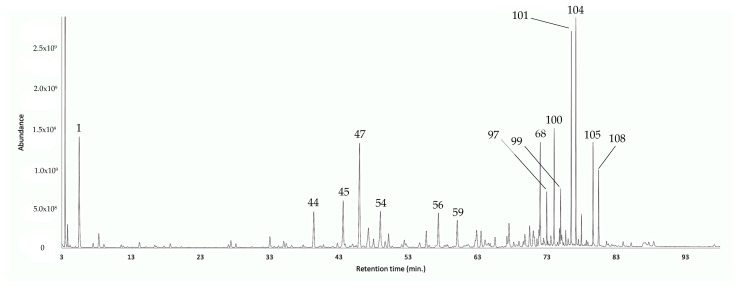
GC–MS chromatogram of the EO from the leaves of *G. rugulosa* in a polyethylene glycol-based column. The main components are represented with the respective peak number, according to Table 1.

According to its chromatographic profiles (Figure 2 and Figure 3), the EO from leaves of *G. rugulosa* was composed of three main groups of components: a poor monoterpene fraction, an important sesquiterpene fraction, and an abundant heavy fraction, characterized by long-chained alcohols and alkanes.

On the one hand, in the monoterpene fraction, α-pinene (peak 1) was the major compound, corresponding to about 5.3–6.0% by weight of the whole EO. On the other hand, the sesquiterpene fraction was dominated (according to the elution order) by (*E*)-β-caryophyllene (peak 44, 2.4–2.8%), α-humulene (peak 45, 3.0–3.2%), germacrene D (peak 47, 4.9–6.5%), δ-cadinene (peak 54, 2.2–2.3%), an unidentified compound of MW 220 (peak 56, 3.0–3.5%), caryophyllene oxide (peak 59, 1.6–2.2%), and α-cadinol (peak 68, 3.8–4.4%), altogether contributing for 20.9–24.9% of the EO mass. Lastly, the heavy fraction mainly constituted 1-nonadecanol (peak 97, 1.7–1.9%), 1-eicosanol (peak 99, 0.9–1.2%), *n*-tricosane (peak 100, 3.3–3.4%), 1-heneicosanol (peak 101, 4.5–5.8%), *n*-pentacosane (peak 104, 5.8–7.1%), 1-tricosanol (peak 105, 4.0–4.5%), and *n*-heptacosane (peak 108, 3.0–3.5%). All these heavy aliphatic metabolites, most likely biosynthetically proceeding from the acetate pathway, accounted for about 23.2–27.4% of the whole EO mass.

### 2.2. Enantioselective Analysis of the EO

For almost all the identified enantiomers, the enantioselective analysis was carried out through a 2,3-diacetyl-6-*tert*-butyldimethylsilyl-β-cyclodextrin capillary column, with the exception of α-copaene and germacrene D. For these compounds, a 2,3-diethyl-6-*tert*-butyldimethylsilyl-β-cyclodextrin column was used since their enantiomers are inseparable with the other chiral selector. As a result, eight enantiomeric pairs and two enantiomerically pure terpenes were detected. On the one hand, most of the chiral metabolites were present as scalemic mixtures, whereas α-terpineol was practically a racemate. On the other hand, (1*R*,5*R*)-(+)-β-pinene and (*S*)-(+)-β-phellandrene were enantiomerically pure. All the enantiomers were identified through the MS spectrum and by comparing their linear retention indices (LRI) with those of a mixture of enantiomerically pure standards. The enantiomeric distribution and the enantiomeric excess (*e.e.*) of the detected enantiomers are reported in Table 2.

## 3. Discussion

### 3.1. Chemical Composition and Main Components

The chemical analyses were carried out through two orthogonal columns, affording reciprocally consistent results. Most of the components identified through the nonpolar column were confirmed on the polar one, with few exceptions for some minority compounds. According to our experience, this is not an unusual phenomenon, due to the higher baseline that is sometimes observed with polyethylene glycol stationary phases. As a result, the total quantitative analysis on the nonpolar column resulted a little higher than the one with the polar stationary phase (93.0% vs. 87.3%). This discrepancy is actually acceptable if we consider that it was a 6% difference, spread over 112 compounds. On the other hand, the polar column permitted to separate some constituents that were physically inseparable with the nonpolar phase. Among them, a major compound, corresponding to peak 99, was included.

### 3.2. Chemical Composition and Main Components

As previously mentioned in Section 2.1, the EO distilled from the leaves of *G. rugulosa* can be described as composed of three main fractions: a monoterpene fraction, a sesquiterpene fraction, and a heavy fraction, the latter constituting long-chained alcohols and alkanes. This last fraction, despite being very abundant, is not common in most EOs, and its constituents are not known for presenting interesting biological activities or constituting important toxicological issues. For these reasons, the discussion of the present volatile fraction focuses on its terpene components. With this respect, the chemical composition of this EO is coherent with the one discussed, in a previous paper, for the entire genus *Gynoxys* and especially for the species *G. miniphylla* [18]. In fact, we can find many common major components, which can be better visualized normalizing each amount to the only terpene fraction, in order to neglect the contribution of the heavy components. The results of this approach are shown in Table 3.

It can be observed that these two EOs share, with the same order of magnitude, α-pinene, (*E*)-β-caryophyllene, α-humulene, germacrene D, δ-cadinene, and α-cadinol, whereas α-phellandrene and β-phellandrene are only typical of *G. miniphylla*. Furthermore, on the one hand, *trans*-myrtanol acetate is only present in *G. miniphylla*, whereas, on the other hand, caryophyllene oxide was only detected in *G. rugulosa*.

### 3.3. Biological Activities of Major Components

According to the chemical composition, we could hypothetically expect for *G. rugulosa* EO some of the properties expectable for the volatile fraction of *G. miniphylla*. For example, due to the high amount of α-pinene, the anti-inflammatory, bronchodilator, antibacterial, antifungal, and antileishmanial activities must be considered [92,93,94,95,96,97]. Likewise, a potential cholinergic capacity could be expected [98,99].

For what concerns germacrene D, to the best of the authors’ knowledge, no important biological activities have been described in the literature. This sesquiterpene is mainly known for its ecological role as an attractive for the moths of genus *Heliothis* and *Helicoverpa* [100,101,102].

Another important component is (*E*)-β-caryophyllene, which is probably the most common sesquiterpene hydrocarbon in EOs. This metabolite is known to possess a very wide range of biological activities, such as neuroprotective, anti-inflammatory, sedative, anxiolytic, antidepressant, anticonvulsant, and antitumor. Despite the most important activity probably being the anti-inflammatory one, exerted by (*E*)-β-caryophyllene via countless different mechanisms, this metabolite became quite known for being a non-psychogenic selective agonist of type 2 cannabinoid receptors (CB2-R) [103].

Another major component is α-humulene, relatively more abundant than (*E*)-β-caryophyllene in this EO. This metabolite is biogenetically related to (*E*)-β-caryophyllene, and that is the reason why we often found both sesquiterpenes together in many EOs. Like (*E*)-β-caryophyllene, the very common α-humulene has also been the object of pharmacological studies [104]. The main biological activity reported for α-humulene is its anticancer property, which it shares with its isomer (*E*)-β-caryophyllene. Furthermore, α-humulene also synergically enhances the antitumor activity of typical cytotoxic drugs (e.g., paclitaxel), by increasing their bioavailability. Anti-inflammatory, antimicrobial, antileishmanial, antiparasitic, cicatrizing, and gastroprotective activities, among others, have also been demonstrated. Of all these latter activities, the anti-inflammatory one is probably the most promising [104].

Another very common but quite less studied sesquiterpene is δ-cadinene. This metabolite is very abundant in some EOs, such as the one obtained from *Kadsura longipedunculata* (21.8%) and *Cedar atlantica* (36.3%) [105,106]. According to the literature, both EOs presented a strong antioxidant and antibacterial activity against Gram-positive bacteria. In addition, on the one hand, the EO from *K. longipedunculata* demonstrated a potential in vitro anti-inflammatory activity, a pro-apoptosis capacity, and a poor cytotoxic activity [105]. On the other hand, the EO from *C. atlantica* was mainly interesting for its anti-insect and antibiofilm activities [106].

Lastly, an interesting biological property must be mentioned for α-cadinol. In 2007, Wen et al. investigated the antiviral activity of more than 200 natural products against the severe acute respiratory syndrome coronavirus (SARS-CoV). Of all the assayed products, only 22 showed a strong activity; α-cadinol was among them [107].

### 3.4. Significance of the Enantiomeric Composition

The description of the enantiomeric profile for a new EO is currently a key aspect of its chemical analysis. The importance of the enantioselective analysis is evident if we consider that two enantiomers, chemically indistinguishable in a nonchiral medium, usually show dramatically different in vivo biological properties. In particular, the optical isomers can present different olfactory properties. For this reason, two EOs, showing a very similar chemical composition, can be characterized by two completely different aromas [108]. This phenomenon cannot be explained by a classical chemical analysis but can be understand comparing the enantioselective profiles.

Comparing the EO from *G. rugulosa* with the volatile fraction of *G. miniphylla*, the two enantiomeric profiles appear dramatically different [18]. This variability, which can also depend on ecological and climatic factors, attests to the existence in plants of different biosynthetic pathways, where diverse enzymes catalyze the synthesis of different enantiomers for possibly different functions.

## 4. Materials and Methods

### 4.1. Plant Material

The leaves of *G. rugulosa* were collected on 29 July 2020, from many shrubs in the range of 200 m around a central point of coordinates 03°59′22″ S and 79°08′41″ W, at an altitude of 2820–2900 m above the sea level. After collection, the leaves were dried at 35 °C for 48 h and stored in a dark fresh place until use. The plant was identified by one of the authors (N.C.), and a botanical specimen was deposited at the herbarium of the UTPL, with voucher code 14664. The identification was carried out on the basis of the voucher with code MO-1891627/A:4813456, deposited at the herbarium of the Missouri Botanical Garden, Saint Louis, MO, USA. This investigation was carried out under permission of the Ministry of Environment, Water, and Ecological Transition of Ecuador, with MAATE registry number MAE-DNB-CM-2016-0048.

### 4.2. EO Distillation and Sample Preparation

**Figure 4:**
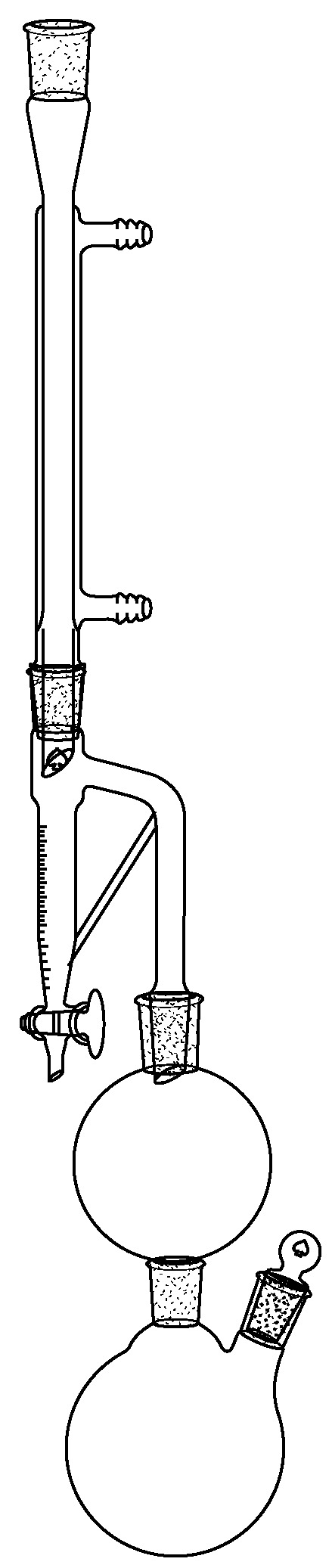
Distillation apparatus used in this study.

The dry, whole leaves were analytically steam-distilled in a glass Marcusson-type apparatus, where the plant material was placed in a separated reservoir, installed between the water heater and the condenser. The bottom of the collection tube was connected to the vapor conduct, such that the aqueous phase was recycled during the process (see Figure 4). Moreover, the collection tube was refrigerated, to avoid overheating of the EO. A volume of 2 mL of cyclohexane, containing *n*-nonane as an internal standard (0.70 mg/mL), was placed over the aqueous phase in the collection tube. With this configuration, the condensed vapors passed through the cyclohexane layer before collection, and the EO was concentrated in the organic phase. The distillation was repeated four times, for 4 h each, obtaining four samples of EO in cyclohexane, which were directly injected into GC (injection volume: 1 μL). The four distillations were carried out with 50.3 g, 33.4 g, 33.2 g, and 34.5 g of dry leaves respectively.

### 4.3. Qualitative (GC–MS) and Quantitative (GC–FID) Chemical Analyses

The qualitative analysis of *G. rugulosa* EO was carried out with gas chromatography–mass spectrometry (GC–MS) equipment, consisting of a Trace 1310 gas chromatograph, coupled to a simple quadrupole mass spectrometry detector, model ISQ 7000 (Thermo Fisher Scientific, Walthan, MA, USA). The mass spectrometer was operated in SCAN mode (scan range 40–400 *m*/*z*), with the electron ionization (EI) source set at 70 eV, the ion source at 230 °C, and the transfer line at 200 °C. A nonpolar column, based on 5% phenyl-methylpolysiloxane, and a polar one, based on a polyethylene glycol stationary phase, were applied to both the qualitative and the quantitative analyses. The nonpolar column was DB-5ms (30 m long, 0.25 mm internal diameter, and 0.25 μm film thickness), whereas the polar one was HP-INNOWax (30 m × 0.25 mm × 0.25 μm), both purchased from Agilent Technology (Santa Clara, CA, USA). For the nonpolar column, the GC oven was operated according to the following program: 50 °C for 10 min., followed by a first thermal gradient of 2 °C/min until 170 °C, and then a second gradient of 10 °C/min until 250 °C, which was maintained for 20 min (total time 98 min). With the polar column, the same thermal program was applied, except that the final temperature was set at 230 °C, due to the lower stability of the polyethylene glycol stationary phase. The injector was operated in split mode (40:1), and its temperature was set at 230 °C. The carrier gas (GC grade helium, from Indura, Guayaquil, Ecuador) was maintained at a constant flow of 1 mL/min. The components of the EO were identified by calculating the linear retention indices (LRIs) according to Van den Dool and Kratz, and by comparing these values and the respective mass spectra with data from literature (see Table 1) [109].

The quantitative analysis was conducted with the same instrument, equipped with a flame ionization detector (FID), and the same two columns used for the qualitative one. The injector parameters, carrier gas flow, and thermal programs were the same as the GC–MS analyses, except for the final temperature time, which was set at 30 min. The constituents of the EO were quantified by external calibration, using *iso*-propyl caproate as the calibration standard and *n*-nonane as the internal standard. A six-point calibration curve was traced for each column, as previously described in the literature, with a correlation coefficient of 0.998 [16]. The use of *iso*-propyl caproate as a quantification standard is based on the principle that, with FID detection, the relative response factors (RRFs) of different analytes versus a unique standard only depend on the combustion enthalpy and, consequently, on the molecular formula of each compound. Therefore, the RRF of each EO component was calculated as described in the literature [110,111]. The total amount of EO, against which the percentage of each component was calculated, was analytically determined through the total area of the chromatogram, to which a mean RRF value was applied. All the analytical-grade solvents, the *n*-alkanes (C_9_–C_30_) for retention indices, and the internal standard (*n*-nonane) were purchased from Sigma-Aldrich (St. Louis, MO, USA). The calibration standard was isopropyl caproate, obtained via synthesis in the authors’ laboratory and purified to 98.8% (GC–FID).

### 4.4. Enantioselective Analyses

The enantioselective analyses were carried out by GC–MS, through two enantioselective capillary columns. They were based on 2,3-diethyl-6-*tert*-butyldimethylsilyl-β-cyclodextrin and 2,3-diacetyl-6-*tert*-butyldimethylsilyl-β-cyclodextrin as chiral selectors (25 m × 250 μm internal diameter × 0.25 μm phase thickness, from Mega, MI, Italy). The GC–MS was operated with the same injector and MS parameters of the qualitative ones. With both enantioselective columns, the following thermal program was applied: 50 °C for 1 min, followed by a thermal gradient of 2 °C/min until 220 °C, which was maintained for 10 min (total time 96 min). Unlike the qualitative and quantitative analyses, a carrier gas constant pressure of 70 kPa was used instead of the constant flow of 1 mL/min. The enantiomers present in the EO, which were separable on the chiral selectors, were identified through the injection of enantiomerically pure standards (1 mg/mL, split 40:1, 1 μL injected). In this case, a mixture of *n*-alkanes (C_9_–C_21_) was also injected to calculate the retention indices.

## 5. Conclusions

The leaves of the Andean species *Gynoxys rugulosa* Muschl. produce an essential oil, whose chemical and enantiomeric composition was described in the present study for the first time. Despite the low distillation yield, this volatile fraction could possess some interesting biological properties, due to its chemical composition. In fact, thanks to the presence of (*E*)-β-caryophyllene, α-humulene, and δ-cadinene, the EO of *G. rugulosa* could be promising as an antibacterial agent against Gram-positive bacteria and as an anti-inflammatory product. Furthermore, the presence of different biosynthetic pathways, selective for the biosynthesis of specific enantiomers, was proposed. The biological activities, suggested in the present work, should be experimentally verified in future.

## Figures and Tables

**Table 1:** Chemical composition of *G. rugulosa* EO through 5% phenyl-methylpolysiloxane and polyethylene glycol GC columns.

N.	Compound	5% Phenyl-Methylpolysiloxane					Polyethylene Glycol				
		LRI ^a^	LRI ^b^	%	σ	Reference	LRI ^a^	LRI ^b^	%	σ	Reference
1	**α-pinene**	933	932	6.0	1.22	[21]	1019	1019	5.3	0.75	[22]
2	sabinene	974	969	0.3	0.03	[21]	1115	1115	0.3	0.03	[23]
3	β-pinene	978	974	1.6	0.37	[21]	1104	1103	1.4	0.20	[24]
4	myrcene	992	988	trace	-	[21]	1162	1162	0.2	0.04	[25]
5	2-pentyl furan	994	984	0.6	0.06	[21]	1231	1230	0.4	0.04	[26]
6	*n*-decane	1000	1000	0.2	0.01	[21]	1000	1000	0.1	0.02	-
7	*trans*-2-(2-pentenyl)-furan	1004	1004			[27]	1302	1282	0.1	0.02	[28]
8	α-phellandrene	1006	1002	0.1	0.02	[21]	1157	1158	0.2	0.04	[29]
9	(2*E*,4*E*)-heptadienal	1008	1005	0.4	0.02	[21]	1485	1488	0.1	0.01	[30]
10	*n*-octanal	1010	998			[21]	1286	1286	0.1	0.02	[31]
11	α-terpinene	1017	1014	trace	-	[21]	1172	1174	0.1	0.02	[32]
12	(2*E*,4*Z*)-heptadienal	1023	1013	0.2	0.02	[33]	1460	1464	0.1	0.04	[34]
13	*p*-cymene	1026	1020	0.4	0.01	[21]	1263	1265	0.3	0.06	[35]
14	limonene	1029	1024	0.7	0.10	[21]	1191	1190	0.1	0.03	[36]
15	β-phellandrene	1031	1025			[21]	1200	1200	0.5	0.09	[37]
16	(*E*)-β-ocimene	1048	1044	trace	-	[21]	1250	1250	0.2	0.03	[38]
17	benzene acetaldehyde	1055	1036	0.3	0.04	[21]	1636	1636	0.3	0.08	[26]
18	terpinolene	1085	1086	0.1	0.02	[21]	1274	1271	0.1	0.02	[39]
19	linalool	1107	1095	0.3	0.10	[21]	1552	1552	0.3	0.07	[40]
20	*n*-nonanal	1113	1100	0.9	0.09	[21]	1389	1389	0.9	0.20	[41]
21	*p*-mentha-1,5-dien-8-ol	1182	1166	0.1	0.16	[21]	1722	1723	0.1	0.06	[42]
22	terpinen-4-ol	1187	1174			[21]	1593	1594	0.1	0.03	[43]
23	octanoic acid	1190	1190	0.1	0.12	[44]	-	-	-	-	-
24	*p*-cymen-8-ol	1198	1179	0.3	0.06	[21]	1845	1845	0.3	0.09	[45]
25	cryptone	1199	1183			[21]	1647	1644	2.4	2.09	[46]
26	α-terpineol	1204	1186	0.2	0.08	[21]	1690	1689	0.4	0.10	[47]
27	*n*-decanal	1214	1201	0.3	0.03	[21]	1494	1492	0.2	0.12	[48]
28	verbenone	1220	1204	0.2	0.07	[21]	-	-	-	-	-
29	pulegone	1228	1233	trace	-	[21]	-	-	-	-	-
30	*trans*-carveol	1230	1215	trace	-	[21]	1829	1830	0.1	0.02	[49]
31	nerol	1233	1227	trace	-	[21]	1755	1755	0.3	0.20	[50]
32	*trans*-chrysanthenyl acetate	1234	1235	trace	-	[21]	-	-	-	-	-
33	geraniol	1260	1249	trace	-	[21]	1847	1847	0.2	0.06	[51]
34	(2*E*)-decenal	1272	1260	0.3	0.06	[21]	1634	1634	0.3	0.06	[52]
35	nonanoic acid	1287	1267	0.3	0.20	[21]	2126	2124	0.1	0.06	[53]
36	*p*-vinylguaiacol	1323	1309	0.5	0.37	[21]	2190	2190	0.9	0.13	[54]
37	(2*E*,4*E*)-decadienal	1331	1315	trace	-	[21]	1780	1780	0.1	0.04	[55]
38	α-copaene	1375	1374	1.2	0.16	[21]	1475	1475	0.9	0.15	[51]
39	(*E*)-β-damascenone	1386	1383	0.8	0.21	[21]	1806	1803	0.7	0.09	[56]
40	β-cubebene	1388	1387	0.1	0.05	[21]	1524	1522	0.2	0.06	[36]
41	*n*-tetradecane	1400	1400	0.2	0.03	[21]	1400	1400	0.5	0.36	-
42	α-gurjunene	1406	1409	0.5	0.03	[21]	1506	1507	0.5	0.10	[57]
43	4-(2,4,4-trimethylcyclohexa-1,5-dienyl)-but-3-en-2-one	1416	1423	0.1	0.18	[58]	-	-	-	-	-
44	**(*E*)-β-caryophyllene**	1420	1417	2.8	0.45	[21]	1576	1575	2.4	0.54	[51]
45	**α-humulene**	1457	1452	3.2	0.54	[21]	1648	1649	3.0	0.66	[36]
46	γ-muurolene	1477	1478	0.2	0.07	[21]	1672	1675	0.3	0.07	[59]
47	**germacrene D**	1484	1480	6.5	1.71	[21]	1689	1690	4.9	1.49	[29]
48	(*E*)-β-ionone	1487	1487			[21]	1920	1923	0.4	0.14	[39]
49	(Z,E)-α-farnesene	1493	1491	0.6	0.18	[21]	1725	1725	0.9	0.31	[60]
50	α-zingiberene	1497	1493	0.9	0.92	[21]	1711	1713	0.8	0.50	[61]
51	α-muurolene	1501	1500	0.6	0.06	[21]	1709	1706	trace	-	[62]
52	γ-cadinene	1516	1513	0.2	0.15	[21]	1740	1738	trace	-	[63]
53	*n*-tridecanal	1518	1509	0.6	0.06	[21]	1810	1809	trace	-	[64]
54	**δ-cadinene**	1521	1523	2.2	0.49	[21]	1743	1744	2.3	0.76	[45]
55	unidentified (MW = 220)	1530	-	0.7	0.12	[21]	1805	-	0.7	0.10	-
56	**unidentified (MW = 220)**	1548	-	3.5	0.71	[21]	1894	-	3.0	0.62	-
57	germacrene D-4-ol	1583	1574	2.0	0.55	[21]	2033	2038	0.2	0.04	[65]
58	spathulenol	1585	1577			[21]	2105	2106	1.6	0.57	[45]
59	**caryophyllene oxide**	1589	1582	2.2	0.77	[21]	1952	1953	1.6	0.61	[66]
60	*n*-hexadecane	1600	1600	0.1	0.06	[21]	1600	1600	0.2	0.05	-
61	viridiflorol	1601	1592	0.9	0.06	[21]	2084	2084	0.5	0.10	[67]
62	ledol	1611	1602	0.4	0.01	[21]	2004	2007	0.2	0.19	[68]
63	unidentified (MW = 220)	1618	-	1.5	0.68	[21]	2007	-	1.2	0.35	-
64	cubenol	1635	1645	0.2	0.10	[21]	2043	2043	0.1	0.12	[68]
65	*epi*-α-cadinol	1651	1638	0.7	0.54	[21]	2159	2160	1.4	0.54	[49]
66	*epi*-α-muurolol	1653	1640	1.3	0.25	[21]	2174	2172	1.4	0.31	[69]
67	α-muurolol (= torreyol)	1656	1644	0.8	0.18	[21]	2187	2187	0.9	0.27	[70]
68	**α-cadinol**	1666	1652	3.8	0.63	[21]	2217	2218	4.4	0.85	[69]
69	unidentified (MW = 220)	1668	-	1.3	0.55	[21]	2145	-	1.0	0.36	-
70	unidentified (MW = 220)	1670	-			[21]	-	-	-	-	-
71	α-amyl cinnamyl alcohol	1670	1682			[21]	-	-	-	-	-
72	ar-turmerone	1675	1668	0.1	0.06	[21]	-	-	-	-	-
73	khusinol	1681	1679	1.2	0.12	[21]	2423	-	1.1	0.06	§
74	(1*R*,7*S*,*E*)-7-isopropyl-4,10-dimethylenecyclodec-5-enol	1698	1695	1.2	0.33	[71]	-	-	-	-	-
75	unidentified (MW = 220)	1700	-			[21]	1433	-	0.4	0.08	-
76	amorpha-4,9-dien-2-ol	1702	1700	0.3	0.38	[21]	2345	-	0.5	0.26	§
77	*n*-pentadecanal	1724	1717	1.4	0.21	[21]	2021	2020	1.0	0.38	[72]
78	unidentified (MW = 236)	1783	-	0.4	0.13	[21]	-	-	-	-	-
79	*n*-octadecane	1800	1800	trace	-	[21]	1800	1800	0.3	0.15	-
80	14-hydroxy-δ-cadinene	1811	1803	0.1	0.03	[21]	2588	2607	0.2	0.01	[73]
81	*n*-hexadecanal	1828	1822	0.1	0.03	[74]	2129	2132	0.1	0.04	[75]
82	(2*E*,6*E*)-farnesyl acetate	1844	1845	0.2	0.02	[21]	2263	2265	0.5	0.11	[25]
83	6,10,14-trimethyl-2-pentadecanone	1851	1848	0.3	0.02	[76]	2120	2125	0.4	0.11	[77]
84	*n*-hexadecanol	1891	1874	trace	-	[21]	2356	2355	0.7	0.13	[78]
85	9-nonadecene	1893	1893			[79]	-	-	-	-	-
86	*n*-nonadecane	1900	1900	0.1	0.01	[21]	1900	1900	0.3	0.03	-
87	unidentified (MW = 216)	1908	-	trace	-	[21]	-	-	-	-	-
88	(5*E*,9*E*)-farnesyl acetone	1919	1913			[21]	2370	2375	0.3	0.06	[80]
89	*n*-heptadecanal	1929	1930	0.4	0.05	[81]	2238	2247	0.3	0.05	[82]
90	phytol	1950	1942	trace	-	[21]	2612	2611	0.2	0.06	[68]
91	*n*-hexadecanoic acid	1975	1975	0.6	0.11	[83]	2850	2871	0.4	0.09	[84]
92	unidentified (MW = 256)	1979	-	0.3	0.03	[21]	-	-	-	-	-
93	1-heptadecanol	1993	1993	0.2	0.03	[81]	2454	2451	0.1	0.06	[44]
94	*n*-eicosane	2000	2000	0.1	0.01	[21]	2000	2000	0.7	0.46	-
95	1-octadecanol	2093	2077	1.0	0.20	[21]	2553	2558	1.2	0.32	[85]
96	*n*-heneicosane	2100	2100	0.5	0.04	[21]	2100	2100	0.2	0.20	-
97	1-nonadecanol	2196	2195	1.9	0.36	[86]	2654	2646	1.7	1.18	[44]
98	*n*-docosane	2200	2200	0.3	0.17	[21]	2200	2200	0.5	0.33	-
99	1-eicosanol	2296	2292	1.2	0.22	[87]	2724	2717	0.9	0.10	[88]
100	***n*-tricosane**	2300	2300	3.4	0.70	[21]	2300	2300	3.3	0.71	-
101	**1-heneicosanol**	2397	2380	5.8	1.49	[89]	2887	-	4.5	1.30	§
102	*n*-tetracosane	2400	2400	trace	-	[21]	2400	2400	1.1	0.27	-
103	1-docosanol	2495	2493	0.2	0.04	[90]	-	-	-	-	-
104	***n*-pentacosane**	2500	2500	7.1	1.99	[21]	2500	2500	5.8	1.65	-
105	**1-tricosanol**	2598	-	4.5	1.11	§	3566	-	4.0	1.00	§
106	*n*-hexacosane	2600	2600			[21]	2600	2600	0.3	0.09	-
107	*n*-tetracosanal	2644	2650	0.5	0.15	[91]	-	-	-	-	-
108	***n*-heptacosane**	2700	2700	3.5	0.87	[21]	2700	2700	3.0	0.88	-
109	1-pentacosanol	2797	-	0.5	0.11	§	-	-	-	-	-
110	*n*-octacosane	2800	2800			[21]	2800	2800	0.5	0.17	-
111	1-hexacosanol	2860	2862	0.8	0.23	[21]	-	-	-	-	-
112	*n*-triacontane	3000	3000	0.3	0.07	[21]	-	-	-	-	-
	Monoterpene hydrocarbons			9.2					8.7		
	Oxygenated monoterpenes			1.1					4.2		
	Sesquiterpene hydrocarbons			23.2					19.9		
	Oxygenated sesquiterpenes			20.1					19.2		
	Others			39.4					35.3		
	Total			93.0					87.3		

^a^ Calculated linear retention index (LRI); ^b^ reference linear retention index (LRI); trace: <0.1%; §: identified by MS spectrum only; %: percentage amount by GC–FID; σ: standard deviation; MW: molecular weight. The compounds in bold represent the main components (≥2.5% on at least one column).

**Table 2:** Linear retention indices (LRI), enantiomeric distribution (%), and enantiomeric excess (*e.e.*) of some chiral terpenes in *G. rugulosa* leaves EO.

LRI	Enantiomers	Enantiomeric Distribution (%)	*e.e. (%)*
918 *	(1*S*,5*S*)-(−)-α-pinene	62.9	25.8
920 *	(1*R*,5*R*)-(+)-α-pinene	37.1	
972 *	(1*R*,5*R*)-(+)-β-pinene	100.0	100.0
1008 *	(1*R*,5*R*)-(+)-sabinene	72.2	44.4
1016 *	(1*S*,5*S*)-(−)-sabinene	27.8	
1024 *	(*S*)-(+)-α-phellandrene	59.1	18.2
1026 *	(*R*)-(−)-α-phellandrene	40.9	
1075 *	(*S*)-(+)-β-phellandrene	100.0	100.0
1302 *	(*R*)-(−)-linalool	52.4	4.8
1305 *	(*S*)-(+)-linalool	47.6	
1317 **	(1*R*,2*S*,6*S*,7*S*,8*S*)-(−)-α-copaene	4.3	91.4
1319 **	(1*S*,2*R*,6*R*,7*R*,8*R*)-(+)-α-copaene	95.7	
1335 *	(*R*)-(−)-terpinen-4-ol	42.6	14.8
1380 *	(*S*)-(+)-terpinen-4-ol	57.4	
1396 *	(*S*)-(−)-α-terpineol	50.1	0.2
1401 *	(*R*)-(+)-α-terpineol	49.9	
1454 **	(*R*)-(+)-germacrene D	95.5	91.0
1462 **	(*S*)-(−)-germacrene D	4.5	

* 2,3-Diacetyl-6-tert-butyldimethylsilyl-β-cyclodextrin column; ** 2,3-diethyl-6-tert-butyldimethylsilyl-β-cyclodextrin column.

**Table 3:** Normalized abundance of major components in the EOs of *G. rugulosa* and *G. miniphylla*.

Compound	Normalized %	
	*G. rugulosa*	*G. miniphylla*
α-pinene	10.7	15.3
α-phellandrene	0.3	17.6
β-phellandrene	0.9	3.2
*trans*-myrtanol acetate	-	9.3
(*E*)-β-caryophyllene	4.9	2.7
α-humulene	5.9	2.0
germacrene D	11.7	14.8
δ-cadinene	4.2	4.6
caryophyllene oxide	3.9	-
α-cadinol	7.7	2.6

## Data Availability

Raw data are available from the authors (Y.E.M.).
